# 3-Hy­droxy­anilinium *p*-toluene­sulfonate

**DOI:** 10.1107/S1600536813018692

**Published:** 2013-07-20

**Authors:** Impichira Pykkat Bincy, Rengasamy Gopalakrishnan

**Affiliations:** a Department of Physics, Anna University, Chennai 600 025, India

## Abstract

The asymmetric unit of the title salt, C_6_H_8_NO^+^·C_7_H_7_O_3_S^−^, contains two cations and two anions. In the crystal, the cations and anions are linked through extensive N—H⋯O and O—H⋯O hydrogen-bonding inter­actions, which result in *R*_4_^4^(18) and *R*_2_^1^(4) ring motifs, forming a three-dimensional network.

## Related literature

For related structures of 4-toluene­sulfonate salts, see: Koshima *et al.* (2004 ▶); Biradha & Mahata (2005 ▶); Sivakumar *et al.* (2012 ▶).
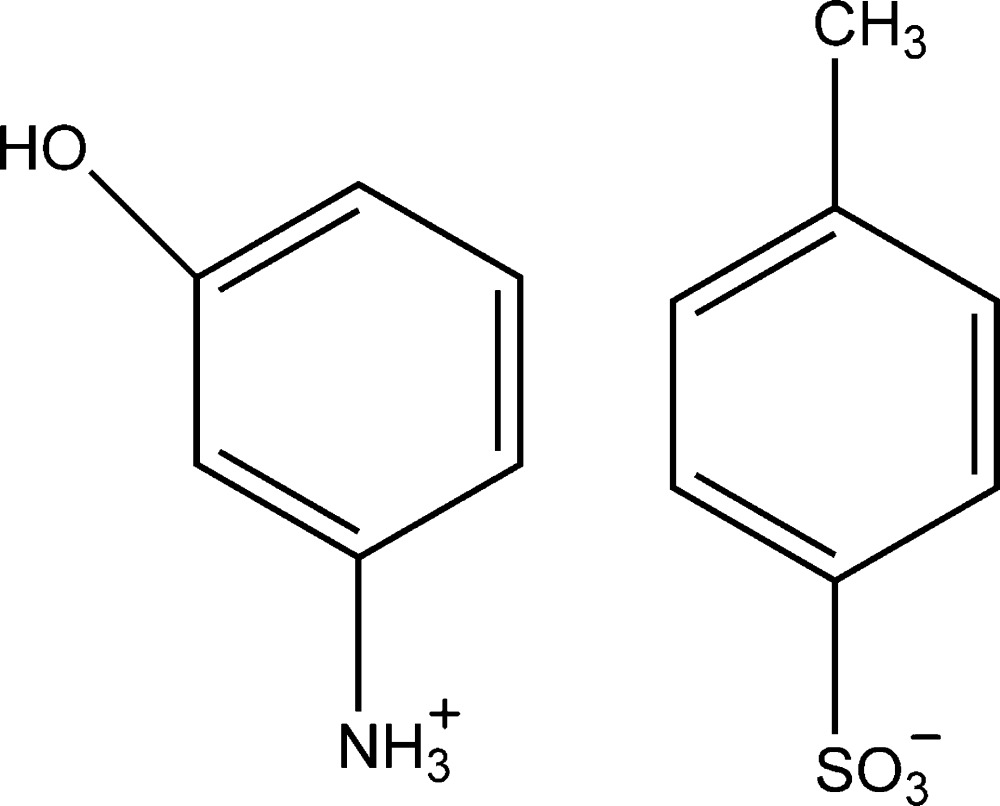


## Experimental

### 

#### Crystal data

C_6_H_8_NO^+^·C_7_H_7_O_3_S^−^
*M* *_r_* = 281.32
Triclinic, 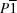

*a* = 9.5775 (3) Å
*b* = 10.8224 (3) Å
*c* = 14.1445 (4) Å
α = 96.787 (2)°
β = 109.701 (1)°
γ = 91.324 (2)°
*V* = 1367.50 (7) Å^3^
*Z* = 4
Mo *K*α radiation
μ = 0.25 mm^−1^
*T* = 293 K
0.30 × 0.25 × 0.20 mm

#### Data collection

Bruker SMART APEXII area-detector diffractometer
Absorption correction: multi-scan (*SADABS*; Bruker, 2008 ▶) *T*_min_ = 0.910, *T*_max_ = 0.953
32207 measured reflections
8651 independent reflections
6679 reflections with *I* > 2σ(*I*)
*R*_int_ = 0.031

#### Refinement

*R*[*F* ^2^ > 2σ(*F* ^2^)] = 0.040
*wR*(*F* ^2^) = 0.122
*S* = 1.03
8651 reflections
372 parameters
6 restraints
H atoms treated by a mixture of independent and constrained refinement
Δρ_max_ = 0.35 e Å^−3^
Δρ_min_ = −0.33 e Å^−3^

### 

Data collection: *APEX2* (Bruker, 2008 ▶); cell refinement: *SAINT* (Bruker, 2008 ▶); data reduction: *SAINT*; program(s) used to solve structure: *SHELXS97* (Sheldrick, 2008 ▶); program(s) used to refine structure: *SHELXL97* (Sheldrick, 2008 ▶); molecular graphics: *ORTEP-3 for Windows* (Farrugia, 2012 ▶); software used to prepare material for publication: *SHELXL97* and *PLATON* (Spek, 2009 ▶).

## Supplementary Material

Crystal structure: contains datablock(s) global, I. DOI: 10.1107/S1600536813018692/kp2454sup1.cif

Structure factors: contains datablock(s) I. DOI: 10.1107/S1600536813018692/kp2454Isup2.hkl

Supplementary material file. DOI: 10.1107/S1600536813018692/kp2454Isup3.cml

Additional supplementary materials:  crystallographic information; 3D view; checkCIF report

## Figures and Tables

**Table 1:** Hydrogen-bond geometry (Å, °)

*D*—H⋯*A*	*D*—H	H⋯*A*	*D*⋯*A*	*D*—H⋯*A*
O7—H7⋯O6^i^	0.82	1.95	2.7657 (16)	173 (2)
N2—H2*C*⋯O4^i^	0.89 (1)	2.06 (1)	2.9441 (18)	170 (2)
N2—H2*B*⋯O3^i^	0.89 (1)	2.31 (2)	2.9020 (17)	124 (2)
N2—H2*A*⋯O3^ii^	0.90 (1)	1.89 (1)	2.7784 (18)	170 (2)
N2—H2*B*⋯O5^ii^	0.89 (1)	2.20 (2)	2.9395 (19)	141 (2)
N1—H1*B*⋯O5^iii^	0.89 (1)	2.15 (2)	2.9406 (19)	147 (2)
N1—H1*B*⋯O6^iii^	0.89 (1)	2.32 (2)	3.1087 (19)	147 (2)
N1—H1*C*⋯O2^iv^	0.89 (1)	1.86 (1)	2.7410 (18)	177 (2)
O8—H8⋯O1	0.82	1.94	2.7216 (17)	160
N1—H1*A*⋯O1	0.90 (1)	1.95 (1)	2.8007 (17)	158 (2)

Symmetry codes: (i) 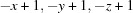; (ii) 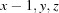; (iii) 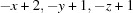; (iv) 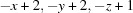.

**Fig. 1.**
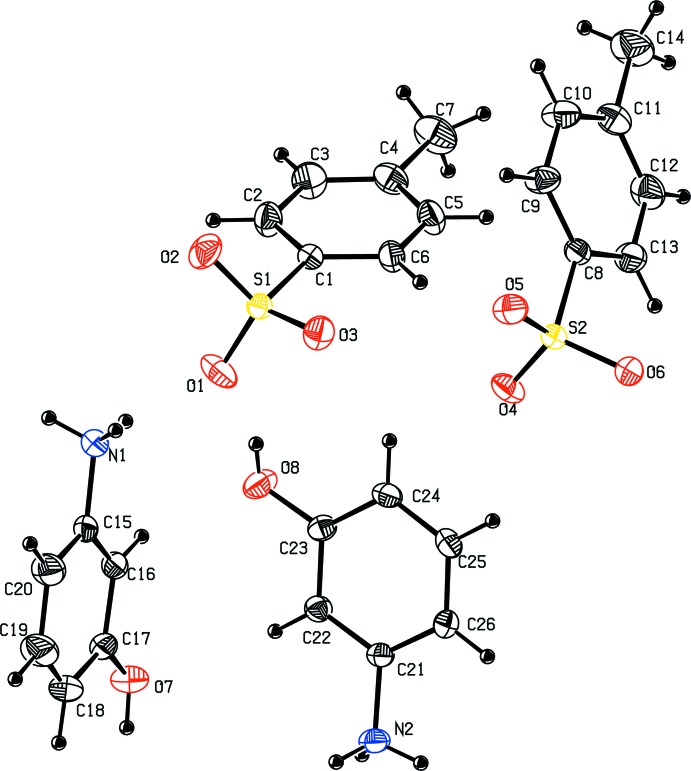
The molecular structure of the title compound, showing displacement ellipsoids drawn at the 30% probability level. H atoms are presented as small spheres of arbitrary radius.

**Fig. 2.**
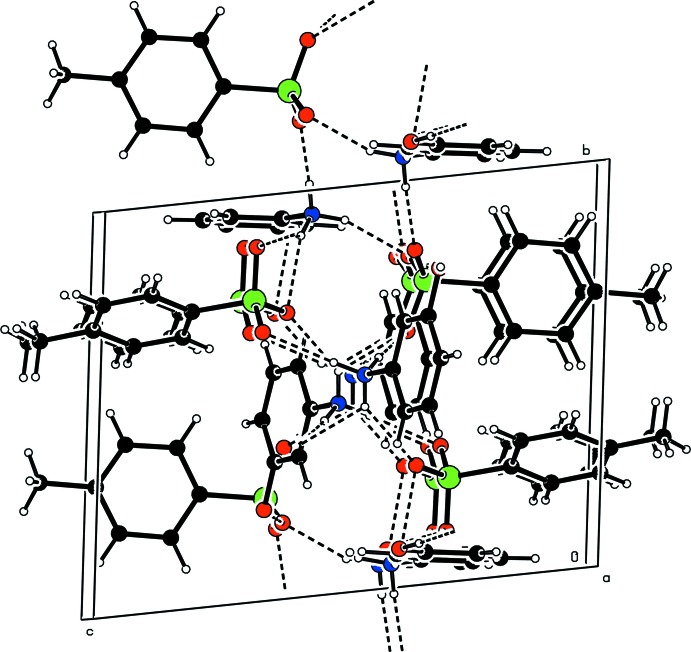
The crystal packing of the title compound viewed down a axis. H-atoms not involved in H-bonds have been excluded for clarity.

## supplementary crystallographic information

### Comment

The asymmetric unit of the title salt (Fig.1) contains two hydroxyanilinium cations [C_6_H_8_N_1_O_1_]^+^ and two 4-toluenesulfonate anions [C_7_H_7_O_3_S_1_]^-^. The aminophenol molecule exists as hydroxyanilinium cation due to the protonation. The 4-toluenesulfonic acid exists as 4-toluenesulfonate due to a proton transfer. The hydroxyl oxygen atoms O7 and O8 attached to the phenyl ring deviate from the ring plane by 0.0528 (1)° and 0.0157 (1)Å, respectively. The crystal packing is stabilsed by intermolcular N—H···O and O—H···O hydrogen bonds (Table 1, Fig.2) involving R^4^_4_(18), R^1^_2_(4) ring motifs.

### Experimental

The title compound was obtained by addition of 3-aminophenol and 4-toluenesulfonic acid in an equimolar ratio using methanol as solvent. After a filtration of resulting solution into a clean beaker, which was covered and kept at room temperature for slow evaporation. After a period of 2 weeks, block-like colourless crystals suitable for X-ray diffraction analysis were obtained.

### Refinement

The H atoms of NH2 groups were located in a difference Fourier map and freely refined. The C-bound H atoms were positioned geometrically and refined using a riding model: C—H = 0.93 and 0.96 Å for CH and CH3 H atoms,respectively, with Uiso(H) = 1.5Ueq(C) for CH3 H atoms and = 1.2Ueq(C) for other H atoms.

### Crystal data

**Table:**

C_6_H_8_NO^+^·C_7_H_7_O_3_S^−^	*Z* = 4
*M**_r_* = 281.32	*F*(000) = 592
Triclinic, *P*1	*D*_x_ = 1.366 Mg m^−^^3^
Hall symbol: -P 1	Mo *K*α radiation, λ = 0.71073 Å
*a* = 9.5775 (3) Å	Cell parameters from 7098 reflections
*b* = 10.8224 (3) Å	θ = 2.3–30.8°
*c* = 14.1445 (4) Å	µ = 0.25 mm^−^^1^
α = 96.787 (2)°	*T* = 293 K
β = 109.701 (1)°	Block, colourless
γ = 91.324 (2)°	0.30 × 0.25 × 0.20 mm
*V* = 1367.50 (7) Å^3^	

### Data collection

**Table:**

Bruker SMART APEXII area-detector diffractometer	8651 independent reflections
Radiation source: fine-focus sealed tube	6679 reflections with *I* > 2σ(*I*)
Graphite monochromator	*R*_int_ = 0.031
ω and φ scans	θ_max_ = 31.0°, θ_min_ = 2.3°
Absorption correction: multi-scan (*SADABS*; Bruker, 2008)	*h* = −13→13
*T*_min_ = 0.910, *T*_max_ = 0.953	*k* = −15→15
32207 measured reflections	*l* = −20→20

### Refinement

**Table:**

Refinement on *F*^2^	Secondary atom site location: difference Fourier map
Least-squares matrix: full	Hydrogen site location: inferred from neighbouring sites
*R*[*F*^2^ > 2σ(*F*^2^)] = 0.040	H atoms treated by a mixture of independent and constrained refinement
*wR*(*F*^2^) = 0.122	*w* = 1/[σ^2^(*F*_o_^2^) + (0.0591*P*)^2^ + 0.346*P*] where *P* = (*F*_o_^2^ + 2*F*_c_^2^)/3
*S* = 1.03	(Δ/σ)_max_ = 0.001
8651 reflections	Δρ_max_ = 0.35 e Å^−^^3^
372 parameters	Δρ_min_ = −0.33 e Å^−^^3^
6 restraints	Extinction correction: *SHELXL97* (Sheldrick, 2008), Fc^*^=kFc[1+0.001xFc^2^λ^3^/sin(2θ)]^-1/4^
Primary atom site location: structure-invariant direct methods	Extinction coefficient: 0.048 (2)

### Special details

**Table:**

Geometry. All esds (except the esd in the dihedral angle between two l.s. planes) are estimated using the full covariance matrix. The cell esds are taken into account individually in the estimation of esds in distances, angles and torsion angles; correlations between esds in cell parameters are only used when they are defined by crystal symmetry. An approximate (isotropic) treatment of cell esds is used for estimating esds involving l.s. planes.
Refinement. Refinement of F^2^ against ALL reflections. The weighted R-factor wR and goodness of fit S are based on F^2^, conventional R-factors R are based on F, with F set to zero for negative F^2^. The threshold expression of F^2^ > 2sigma(F^2^) is used only for calculating R-factors(gt) etc. and is not relevant to the choice of reflections for refinement. R-factors based on F^2^ are statistically about twice as large as those based on F, and R- factors based on ALL data will be even larger.

### Fractional atomic coordinates and isotropic or equivalent isotropic displacement parameters (Å^2^)

**Table:**

	*x*	*y*	*z*	*U*_iso_*/*U*_eq_	
C1	0.79378 (15)	0.72105 (13)	0.22287 (10)	0.0342 (3)	
C2	0.7663 (2)	0.82072 (15)	0.16737 (13)	0.0509 (4)	
H2	0.7882	0.9023	0.1999	0.061*	
C3	0.7056 (2)	0.79676 (18)	0.06283 (13)	0.0595 (5)	
H3	0.6879	0.8634	0.0253	0.071*	
C4	0.67079 (19)	0.67706 (18)	0.01284 (12)	0.0515 (4)	
C5	0.6972 (2)	0.57994 (17)	0.06995 (12)	0.0521 (4)	
H5	0.6734	0.4985	0.0375	0.063*	
C6	0.75833 (18)	0.60085 (14)	0.17446 (11)	0.0435 (3)	
H6	0.7754	0.5341	0.2118	0.052*	
C7	0.6075 (3)	0.6510 (2)	−0.10113 (13)	0.0735 (6)	
H7A	0.5010	0.6440	−0.1227	0.110*	
H7B	0.6424	0.5744	−0.1236	0.110*	
H7C	0.6390	0.7180	−0.1297	0.110*	
C8	0.86225 (15)	0.26820 (13)	0.17460 (10)	0.0367 (3)	
C9	0.97997 (17)	0.32943 (17)	0.16045 (12)	0.0495 (4)	
H9	1.0607	0.3644	0.2160	0.059*	
C10	0.9775 (2)	0.3387 (2)	0.06314 (13)	0.0576 (4)	
H10	1.0577	0.3796	0.0543	0.069*	
C11	0.8593 (2)	0.28898 (18)	−0.02095 (12)	0.0526 (4)	
C12	0.7423 (2)	0.2290 (2)	−0.00521 (13)	0.0596 (5)	
H12	0.6609	0.1954	−0.0608	0.072*	
C13	0.74287 (18)	0.21754 (18)	0.09124 (13)	0.0526 (4)	
H13	0.6630	0.1758	0.1000	0.063*	
C14	0.8580 (3)	0.3018 (3)	−0.12599 (15)	0.0793 (7)	
H14A	0.7661	0.2648	−0.1748	0.119*	
H14B	0.9395	0.2603	−0.1375	0.119*	
H14C	0.8677	0.3886	−0.1326	0.119*	
C15	0.75233 (16)	0.93763 (12)	0.64225 (10)	0.0366 (3)	
C16	0.60302 (16)	0.91742 (13)	0.58994 (11)	0.0371 (3)	
H16	0.5676	0.9027	0.5195	0.044*	
C17	0.50499 (17)	0.91922 (13)	0.64370 (12)	0.0410 (3)	
C18	0.5603 (2)	0.93703 (17)	0.74827 (13)	0.0548 (4)	
H18	0.4956	0.9377	0.7847	0.066*	
C19	0.7110 (2)	0.9538 (2)	0.79838 (13)	0.0652 (5)	
H19	0.7474	0.9644	0.8688	0.078*	
C20	0.8098 (2)	0.95514 (19)	0.74635 (13)	0.0553 (4)	
H20	0.9117	0.9675	0.7806	0.066*	
C21	0.27809 (14)	0.53847 (13)	0.42602 (9)	0.0326 (3)	
C22	0.31807 (15)	0.65362 (13)	0.40693 (10)	0.0362 (3)	
H22	0.2743	0.7247	0.4240	0.043*	
C23	0.42514 (15)	0.66067 (13)	0.36166 (10)	0.0358 (3)	
C24	0.48849 (15)	0.55399 (14)	0.33565 (10)	0.0389 (3)	
H24	0.5606	0.5592	0.3054	0.047*	
C25	0.44440 (15)	0.44001 (14)	0.35467 (11)	0.0395 (3)	
H25	0.4866	0.3685	0.3366	0.047*	
C26	0.33822 (15)	0.43084 (13)	0.40027 (10)	0.0357 (3)	
H26	0.3083	0.3541	0.4132	0.043*	
N1	0.85435 (15)	0.94075 (12)	0.58541 (10)	0.0397 (3)	
N2	0.17042 (14)	0.53203 (13)	0.47848 (10)	0.0419 (3)	
O1	0.76020 (13)	0.81258 (14)	0.38830 (9)	0.0607 (3)	
O2	1.00677 (13)	0.82598 (12)	0.38221 (10)	0.0594 (3)	
O3	0.89749 (14)	0.62868 (11)	0.39024 (8)	0.0508 (3)	
O4	0.74946 (13)	0.33688 (11)	0.31395 (9)	0.0509 (3)	
O5	1.00949 (12)	0.29535 (12)	0.36731 (8)	0.0513 (3)	
O6	0.82415 (14)	0.12609 (10)	0.29910 (9)	0.0522 (3)	
O7	0.35802 (13)	0.90516 (13)	0.58862 (9)	0.0551 (3)	
H7	0.3104	0.8967	0.6262	0.083*	
O8	0.46157 (13)	0.77577 (11)	0.34359 (10)	0.0525 (3)	
H8	0.5470	0.7784	0.3432	0.079*	
S1	0.87120 (4)	0.74922 (3)	0.35574 (2)	0.03467 (9)	
S2	0.86039 (4)	0.25714 (3)	0.29790 (3)	0.03551 (9)	
H1C	0.899 (2)	1.0158 (12)	0.5934 (16)	0.066 (6)*	
H1A	0.811 (2)	0.9178 (19)	0.5186 (8)	0.062 (6)*	
H2A	0.0884 (16)	0.5712 (18)	0.4500 (14)	0.064 (6)*	
H2C	0.206 (2)	0.5702 (19)	0.5421 (9)	0.066 (6)*	
H2B	0.141 (2)	0.4533 (11)	0.4761 (16)	0.069 (6)*	
H1B	0.928 (2)	0.891 (2)	0.6078 (19)	0.092 (8)*	

### Atomic displacement parameters (Å^2^)

**Table:**

	*U*^11^	*U*^22^	*U*^33^	*U*^12^	*U*^13^	*U*^23^
C1	0.0375 (6)	0.0332 (7)	0.0337 (6)	0.0013 (5)	0.0155 (5)	0.0024 (5)
C2	0.0696 (11)	0.0362 (8)	0.0451 (8)	0.0021 (7)	0.0172 (7)	0.0060 (6)
C3	0.0797 (12)	0.0550 (10)	0.0438 (9)	0.0098 (9)	0.0171 (8)	0.0179 (8)
C4	0.0536 (9)	0.0647 (11)	0.0354 (7)	0.0083 (8)	0.0147 (7)	0.0041 (7)
C5	0.0653 (10)	0.0458 (9)	0.0406 (8)	0.0026 (7)	0.0164 (7)	−0.0067 (6)
C6	0.0576 (9)	0.0340 (7)	0.0384 (7)	0.0036 (6)	0.0165 (6)	0.0025 (6)
C7	0.0775 (13)	0.1009 (17)	0.0358 (8)	0.0106 (12)	0.0123 (8)	0.0051 (9)
C8	0.0357 (6)	0.0386 (7)	0.0367 (6)	0.0019 (5)	0.0155 (5)	−0.0004 (5)
C9	0.0382 (7)	0.0692 (11)	0.0396 (7)	−0.0076 (7)	0.0122 (6)	0.0067 (7)
C10	0.0505 (9)	0.0800 (13)	0.0469 (9)	−0.0038 (8)	0.0217 (7)	0.0131 (8)
C11	0.0571 (9)	0.0631 (11)	0.0393 (8)	0.0100 (8)	0.0186 (7)	0.0063 (7)
C12	0.0554 (10)	0.0741 (13)	0.0395 (8)	−0.0054 (9)	0.0092 (7)	−0.0079 (8)
C13	0.0438 (8)	0.0642 (11)	0.0459 (8)	−0.0128 (7)	0.0159 (7)	−0.0069 (7)
C14	0.0955 (16)	0.1021 (18)	0.0428 (10)	0.0034 (14)	0.0267 (10)	0.0109 (10)
C15	0.0476 (7)	0.0280 (6)	0.0378 (7)	0.0018 (5)	0.0197 (6)	0.0031 (5)
C16	0.0465 (7)	0.0315 (7)	0.0371 (7)	0.0051 (5)	0.0184 (6)	0.0072 (5)
C17	0.0502 (8)	0.0330 (7)	0.0472 (8)	0.0023 (6)	0.0250 (6)	0.0096 (6)
C18	0.0688 (11)	0.0593 (10)	0.0480 (9)	−0.0016 (8)	0.0352 (8)	0.0078 (8)
C19	0.0767 (13)	0.0838 (14)	0.0357 (8)	−0.0047 (11)	0.0225 (8)	0.0020 (8)
C20	0.0539 (9)	0.0676 (11)	0.0404 (8)	−0.0054 (8)	0.0141 (7)	−0.0007 (7)
C21	0.0298 (5)	0.0386 (7)	0.0303 (6)	0.0009 (5)	0.0119 (4)	0.0042 (5)
C22	0.0362 (6)	0.0357 (7)	0.0399 (7)	0.0062 (5)	0.0162 (5)	0.0071 (5)
C23	0.0348 (6)	0.0393 (7)	0.0344 (6)	0.0013 (5)	0.0117 (5)	0.0098 (5)
C24	0.0361 (6)	0.0496 (8)	0.0336 (6)	0.0018 (6)	0.0168 (5)	0.0014 (6)
C25	0.0389 (7)	0.0396 (7)	0.0379 (7)	0.0061 (5)	0.0133 (5)	−0.0036 (5)
C26	0.0355 (6)	0.0326 (7)	0.0362 (6)	−0.0015 (5)	0.0097 (5)	0.0024 (5)
N1	0.0427 (6)	0.0377 (7)	0.0415 (6)	0.0027 (5)	0.0189 (5)	0.0026 (5)
N2	0.0398 (6)	0.0464 (8)	0.0474 (7)	0.0022 (5)	0.0244 (6)	0.0089 (6)
O1	0.0479 (6)	0.0841 (9)	0.0462 (6)	0.0099 (6)	0.0192 (5)	−0.0171 (6)
O2	0.0465 (6)	0.0594 (8)	0.0633 (8)	−0.0198 (5)	0.0110 (5)	0.0007 (6)
O3	0.0629 (7)	0.0467 (6)	0.0389 (5)	−0.0019 (5)	0.0112 (5)	0.0101 (5)
O4	0.0560 (6)	0.0547 (7)	0.0506 (6)	0.0180 (5)	0.0296 (5)	0.0042 (5)
O5	0.0436 (6)	0.0681 (8)	0.0385 (5)	−0.0068 (5)	0.0109 (4)	0.0042 (5)
O6	0.0699 (8)	0.0373 (6)	0.0604 (7)	−0.0009 (5)	0.0379 (6)	0.0036 (5)
O7	0.0474 (6)	0.0701 (8)	0.0574 (7)	0.0041 (5)	0.0281 (5)	0.0147 (6)
O8	0.0503 (6)	0.0459 (6)	0.0711 (8)	0.0014 (5)	0.0294 (6)	0.0210 (6)
S1	0.03290 (16)	0.03659 (18)	0.03343 (16)	−0.00323 (12)	0.01232 (12)	−0.00138 (12)
S2	0.03677 (17)	0.03538 (18)	0.03731 (17)	0.00095 (12)	0.01806 (13)	0.00041 (13)

### Geometric parameters (Å, º)

**Table:**

C1—C6	1.374 (2)	C16—H16	0.9300
C1—C2	1.387 (2)	C17—O7	1.3543 (19)
C1—S1	1.7576 (14)	C17—C18	1.380 (2)
C2—C3	1.384 (2)	C18—C19	1.374 (3)
C2—H2	0.9300	C18—H18	0.9300
C3—C4	1.376 (3)	C19—C20	1.381 (3)
C3—H3	0.9300	C19—H19	0.9300
C4—C5	1.377 (3)	C20—H20	0.9300
C4—C7	1.507 (2)	C21—C26	1.3760 (19)
C5—C6	1.382 (2)	C21—C22	1.3783 (19)
C5—H5	0.9300	C21—N2	1.4641 (16)
C6—H6	0.9300	C22—C23	1.3849 (18)
C7—H7A	0.9600	C22—H22	0.9300
C7—H7B	0.9600	C23—O8	1.3617 (17)
C7—H7C	0.9600	C23—C24	1.385 (2)
C8—C9	1.378 (2)	C24—C25	1.380 (2)
C8—C13	1.381 (2)	C24—H24	0.9300
C8—S2	1.7682 (14)	C25—C26	1.3830 (19)
C9—C10	1.384 (2)	C25—H25	0.9300
C9—H9	0.9300	C26—H26	0.9300
C10—C11	1.378 (2)	N1—H1C	0.885 (9)
C10—H10	0.9300	N1—H1A	0.895 (9)
C11—C12	1.377 (3)	N1—H1B	0.893 (10)
C11—C14	1.504 (2)	N2—H2A	0.895 (9)
C12—C13	1.383 (2)	N2—H2C	0.892 (9)
C12—H12	0.9300	N2—H2B	0.886 (9)
C13—H13	0.9300	O1—S1	1.4497 (11)
C14—H14A	0.9600	O2—S1	1.4370 (11)
C14—H14B	0.9600	O3—S1	1.4444 (12)
C14—H14C	0.9600	O4—S2	1.4435 (11)
C15—C16	1.369 (2)	O5—S2	1.4511 (11)
C15—C20	1.375 (2)	O6—S2	1.4558 (11)
C15—N1	1.4617 (18)	O7—H7	0.8200
C16—C17	1.3923 (19)	O8—H8	0.8200
			
C6—C1—C2	120.23 (14)	O7—C17—C16	116.90 (13)
C6—C1—S1	120.08 (11)	C18—C17—C16	119.50 (15)
C2—C1—S1	119.68 (11)	C19—C18—C17	119.91 (15)
C3—C2—C1	118.87 (15)	C19—C18—H18	120.0
C3—C2—H2	120.6	C17—C18—H18	120.0
C1—C2—H2	120.6	C18—C19—C20	121.41 (16)
C4—C3—C2	121.79 (16)	C18—C19—H19	119.3
C4—C3—H3	119.1	C20—C19—H19	119.3
C2—C3—H3	119.1	C15—C20—C19	117.71 (16)
C3—C4—C5	118.09 (15)	C15—C20—H20	121.1
C3—C4—C7	121.74 (17)	C19—C20—H20	121.1
C5—C4—C7	120.17 (17)	C26—C21—C22	122.64 (12)
C4—C5—C6	121.47 (15)	C26—C21—N2	119.15 (12)
C4—C5—H5	119.3	C22—C21—N2	118.18 (12)
C6—C5—H5	119.3	C21—C22—C23	118.24 (13)
C1—C6—C5	119.52 (14)	C21—C22—H22	120.9
C1—C6—H6	120.2	C23—C22—H22	120.9
C5—C6—H6	120.2	O8—C23—C22	116.68 (13)
C4—C7—H7A	109.5	O8—C23—C24	122.94 (12)
C4—C7—H7B	109.5	C22—C23—C24	120.38 (13)
H7A—C7—H7B	109.5	C25—C24—C23	119.83 (12)
C4—C7—H7C	109.5	C25—C24—H24	120.1
H7A—C7—H7C	109.5	C23—C24—H24	120.1
H7B—C7—H7C	109.5	C24—C25—C26	120.79 (13)
C9—C8—C13	119.39 (14)	C24—C25—H25	119.6
C9—C8—S2	120.82 (11)	C26—C25—H25	119.6
C13—C8—S2	119.77 (11)	C21—C26—C25	118.11 (13)
C8—C9—C10	119.68 (15)	C21—C26—H26	120.9
C8—C9—H9	120.2	C25—C26—H26	120.9
C10—C9—H9	120.2	C15—N1—H1C	112.1 (14)
C11—C10—C9	121.79 (16)	C15—N1—H1A	113.8 (13)
C11—C10—H10	119.1	H1C—N1—H1A	106.9 (19)
C9—C10—H10	119.1	C15—N1—H1B	110.6 (17)
C12—C11—C10	117.65 (15)	H1C—N1—H1B	105 (2)
C12—C11—C14	121.49 (18)	H1A—N1—H1B	108 (2)
C10—C11—C14	120.85 (18)	C21—N2—H2A	112.7 (13)
C11—C12—C13	121.62 (16)	C21—N2—H2C	112.2 (14)
C11—C12—H12	119.2	H2A—N2—H2C	104.0 (19)
C13—C12—H12	119.2	C21—N2—H2B	109.9 (14)
C8—C13—C12	119.86 (16)	H2A—N2—H2B	106.4 (19)
C8—C13—H13	120.1	H2C—N2—H2B	111 (2)
C12—C13—H13	120.1	C17—O7—H7	109.5
C11—C14—H14A	109.5	C23—O8—H8	109.5
C11—C14—H14B	109.5	O2—S1—O3	111.93 (8)
H14A—C14—H14B	109.5	O2—S1—O1	112.03 (8)
C11—C14—H14C	109.5	O3—S1—O1	112.64 (8)
H14A—C14—H14C	109.5	O2—S1—C1	108.04 (7)
H14B—C14—H14C	109.5	O3—S1—C1	106.42 (6)
C16—C15—C20	122.37 (14)	O1—S1—C1	105.28 (7)
C16—C15—N1	118.85 (12)	O4—S2—O5	113.19 (7)
C20—C15—N1	118.79 (14)	O4—S2—O6	112.69 (7)
C15—C16—C17	119.05 (13)	O5—S2—O6	110.65 (8)
C15—C16—H16	120.5	O4—S2—C8	107.33 (7)
C17—C16—H16	120.5	O5—S2—C8	106.67 (6)
O7—C17—C18	123.59 (14)	O6—S2—C8	105.79 (7)
			
C6—C1—C2—C3	−1.3 (3)	C17—C18—C19—C20	1.0 (3)
S1—C1—C2—C3	179.86 (14)	C16—C15—C20—C19	−1.0 (3)
C1—C2—C3—C4	0.6 (3)	N1—C15—C20—C19	179.00 (17)
C2—C3—C4—C5	0.4 (3)	C18—C19—C20—C15	−0.8 (3)
C2—C3—C4—C7	−178.76 (19)	C26—C21—C22—C23	−1.2 (2)
C3—C4—C5—C6	−0.7 (3)	N2—C21—C22—C23	176.98 (12)
C7—C4—C5—C6	178.48 (18)	C21—C22—C23—O8	179.78 (12)
C2—C1—C6—C5	1.0 (2)	C21—C22—C23—C24	0.7 (2)
S1—C1—C6—C5	179.85 (13)	O8—C23—C24—C25	−178.89 (13)
C4—C5—C6—C1	0.0 (3)	C22—C23—C24—C25	0.2 (2)
C13—C8—C9—C10	−0.4 (3)	C23—C24—C25—C26	−0.5 (2)
S2—C8—C9—C10	−178.85 (14)	C22—C21—C26—C25	0.9 (2)
C8—C9—C10—C11	0.5 (3)	N2—C21—C26—C25	−177.28 (12)
C9—C10—C11—C12	0.0 (3)	C24—C25—C26—C21	0.0 (2)
C9—C10—C11—C14	179.1 (2)	C6—C1—S1—O2	126.15 (13)
C10—C11—C12—C13	−0.6 (3)	C2—C1—S1—O2	−54.99 (14)
C14—C11—C12—C13	−179.7 (2)	C6—C1—S1—O3	5.78 (14)
C9—C8—C13—C12	−0.2 (3)	C2—C1—S1—O3	−175.36 (13)
S2—C8—C13—C12	178.26 (15)	C6—C1—S1—O1	−113.99 (13)
C11—C12—C13—C8	0.7 (3)	C2—C1—S1—O1	64.87 (14)
C20—C15—C16—C17	2.5 (2)	C9—C8—S2—O4	108.94 (14)
N1—C15—C16—C17	−177.51 (13)	C13—C8—S2—O4	−69.52 (15)
C15—C16—C17—O7	176.70 (13)	C9—C8—S2—O5	−12.66 (16)
C15—C16—C17—C18	−2.2 (2)	C13—C8—S2—O5	168.88 (13)
O7—C17—C18—C19	−178.30 (17)	C9—C8—S2—O6	−130.52 (14)
C16—C17—C18—C19	0.5 (3)	C13—C8—S2—O6	51.02 (15)

### Hydrogen-bond geometry (Å, º)

**Table:**

*D*—H···*A*	*D*—H	H···*A*	*D*···*A*	*D*—H···*A*
O7—H7···O6^i^	0.82	1.95	2.7657 (16)	173 (2)
N2—H2*C*···O4^i^	0.89 (1)	2.06 (1)	2.9441 (18)	170 (2)
N2—H2*B*···O3^i^	0.89 (1)	2.31 (2)	2.9020 (17)	124 (2)
N2—H2*A*···O3^ii^	0.90 (1)	1.89 (1)	2.7784 (18)	170 (2)
N2—H2*B*···O5^ii^	0.89 (1)	2.20 (2)	2.9395 (19)	141 (2)
N1—H1*B*···O5^iii^	0.89 (1)	2.15 (2)	2.9406 (19)	147 (2)
N1—H1*B*···O6^iii^	0.89 (1)	2.32 (2)	3.1087 (19)	147 (2)
N1—H1*C*···O2^iv^	0.89 (1)	1.86 (1)	2.7410 (18)	177 (2)
O8—H8···O1	0.82	1.94	2.7216 (17)	160
N1—H1*A*···O1	0.90 (1)	1.95 (1)	2.8007 (17)	158 (2)

Symmetry codes: (i) −*x*+1, −*y*+1, −*z*+1; (ii) *x*−1, *y*, *z*; (iii) −*x*+2, −*y*+1, −*z*+1; (iv) −*x*+2, −*y*+2, −*z*+1.
